# Les corps étrangers trachéobronchiques chez l'adulte

**DOI:** 10.11604/pamj.2014.19.220.4952

**Published:** 2014-10-28

**Authors:** Hanane Benjelloun, Nahid Zaghba, Abdelaziz Bakhatar, Najiba Yassine, Abdelkrim Bahlaoui

**Affiliations:** 1 Service des Maladies Respiratoires, Centre Hospitalier Ibn Rochd, Casablanca, Maroc

**Keywords:** Corps étranger, bronches, syndrome de pénétration, radiographie thoracique, bronchoscopie, foreign bodies, bronchi, penetration syndrome, chest X-ray, bronchoscopy

## Abstract

L'inhalation d'un corps étranger (CE) est une urgence médicale courante dans tous les âges. Chez l'adulte, cet accident est beaucoup plus rare que chez l'enfant et survient le plus souvent sur des terrains prédisposés. L'objectif est d’évaluer l'approche diagnostique et thérapeutique de l'inhalation endobronchique d'un CE chez l'adulte. Nous rapportons une étude rétrospective réalisée sur une période de plus de 20 ans (entre Janvier 1994 et Mai 2014) concernant 51 cas de CE endobronchiques de l'adulte. Leur mode de révélation, leurs caractéristiques cliniques, radiologiques et évolutives, la nature de ces CE inhalés sont décrits. Il s'agissait de 8 hommes et 43 femmes dont l’âge moyen était de 28 ans. Un terrain prédisposant est noté dans 13 cas. Le syndrome de pénétration était le maitre symptôme révélateur, retrouvé dans 46 cas. Le siège du CE était bronchique droit dans 30 cas. L'extraction est réalisée par bronchoscopie souple dans 40 cas, rigide dans 3 cas et par chirurgie dans 4 cas. Quatre patientes ont rejeté spontanément le corps étranger. La nature du CE était variable mais restait largement dominée par les épingles à foulards. Nous insistons sur la rareté et la gravité potentielle de l'inhalation de CE qui reste dominée dans notre contexte culturel par l’épingle à foulard chez les jeunes filles. En dehors d'un syndrome de pénétration évident, le diagnostic est souvent difficile faisant recours à la bronchoscopie à la fois diagnostique et thérapeutique. Les mesures préventives restent le meilleur traitement.

## Introduction

Les corps étrangers (CE) trachéobronchiques par inhalation accidentelle sont des incidents graves et particulièrement fréquents chez les enfants de bas âge. Chez l'adulte, ils sont moins fréquents, très souvent méconnus et de ce fait de diagnostic souvent difficile. Une extraction endoscopique précoce s'impose afin d’éviter de lourdes conséquences faisant recours parfois à des exérèses pulmonaires délabrantes.

## Méthodes

C'est une étude analytique descriptive rétrospective portant sur 51 cas de corps étrangers endobronchiques de l'adulte colligés entre Janvier 1994 et Mai 2014 au service des maladies respiratoires au CHU Ibn Rochd de Casablanca.

## Résultats

**Figure 1:**
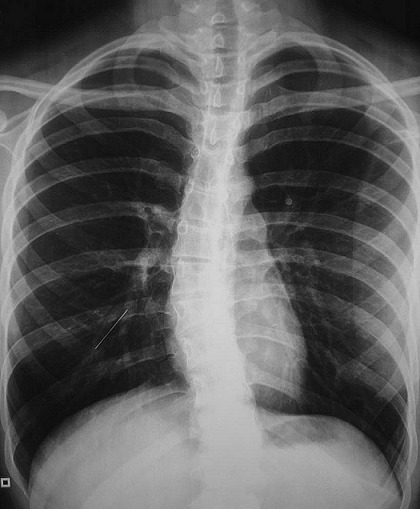
Radiographie thoracique de face montrant une opacité linéaire au niveau de la pyramide basale droite, correspondant à une épingle à foulard

**Figure 2:**
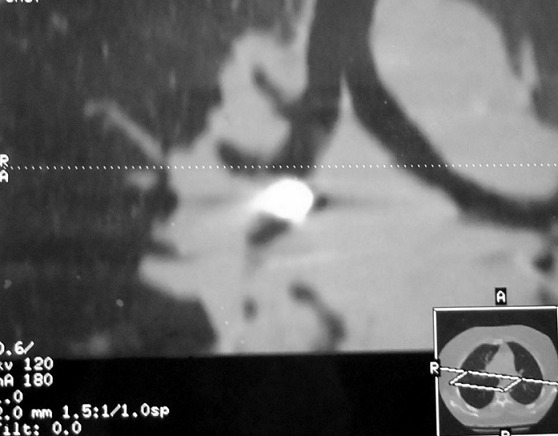
TDM thoracique (coupe coronale) montrant à une formation très dense lobaire inférieure droite, correspondant à une prothèse dentaire

**Figure 3:**
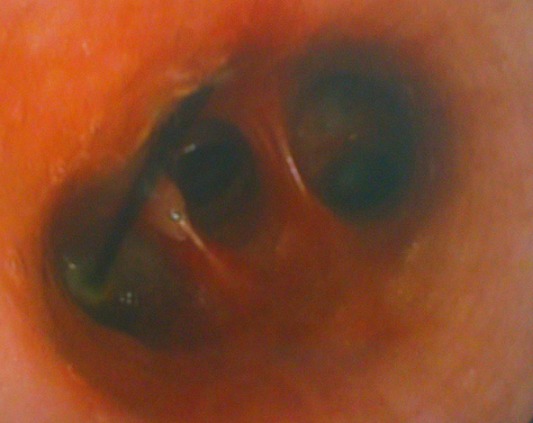
Bronchoscopie souple montrant une épingle à foulard enclavée dans la muqueuse

**Figure 4:**
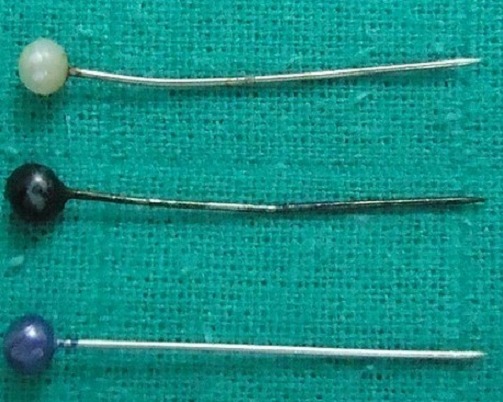
Corps étrangers correspondant à des épingles à foulard extradites

**Figure 5:**
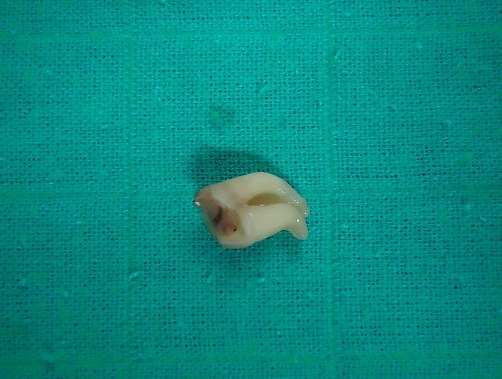
Corps étranger correspondant à une dent extraite

Il s'agissait de 43 femmes et huit hommes. La moyenne d’âge de nos patients était de 28 ans avec des extrêmes allant de 12 à 80 ans. Dans les antécédents, on a retrouvé six cas de patients suivis pour une pathologie neurologique à type d’épilepsie, de syndrome de Guillain barré et de sclérose latérale amyotrophique, deux cas pour dépression, deux autres porteurs de scoliose. La trachéotomie après laryngectomie totale, le traumatisme facial et l'asthme ont été notés dans un cas chacun. Le délai moyen de consultation était de huit jours (0-2 mois). Le motif de consultation était dominé par le syndrome de pénétration dans 46 cas (90%), les infections respiratoires à répétition dans neuf cas et les hémoptysies dans six cas. L'examen clinique était normal dans tous les cas. L'imagerie thoracique (Figure 1, Figure 2) avait montré un CE radio-opaque dans 41 cas (80%) et un foyer d'atélectasie dans quatre cas. Le siège de l'anomalie était bronchique droit dans 30 cas (58,8%), bronchique gauche dans 15 cas (29,4%) et au niveau de la trachée dans six cas (11,7%). Une prémédication systématique à base d'une antibiothérapie (amoxicilline-acide clavulanique (3 g/j)) associée à une corticothérapie orale de courte durée (prédnisolone 1 mg/kg/j) était prescrite dans tous les cas. Une patiente avait rejeté spontanément le CE quelques heures après son hospitalisation avant toute tentative d'extraction endoscopique. La bronchoscopie souple, réalisée de première intention chez nos patients restants, avait retrouvé un état inflammatoire bronchique dans 40 cas, un granulome inflammatoire dans 10 cas, et avait visualisé un CE dans 48 cas (Figure 3), au niveau de la trachée dans cinq cas (9,8%), à droite: la bronche principale dans cinq cas (9,8%), le tronc intermédiaire dans six cas (11,7%) et au niveau de la pyramide basale dans 17 cas (33,3%); à gauche: la bronche principale dans trois cas (5,8%), au niveau de l'orifice segmentaire de la lingula dans un cas (2%) et au niveau de la pyramide basale dans 11 cas (21,5%). L'extraction du CE était réalisée par une bronchoscopie souple dans 41 cas (80,3%) (Figure 4, Figure 5), après seulement une tentative dans 18 cas (35,3%), après une 2ème tentative dans 12 cas (23,5%), une 3ème tentative dans 10 cas (19,6%); par bronchoscopie rigide dans trois cas et par chirurgie dans 4 cas (7,8%). Trois patientes (5,8%) avaient spontanément rejeté le CE après échec de l'extraction endoscopique. La nature du CE (Tableau 1) était variable mais est restée largement dominée dans notre contexte par les épingles à foulard notamment chez les jeunes filles. Le délai moyen d'extraction du CE était de six jours avec des extrêmes allant de quelques heures à trois mois. L’évolution immédiate était bonne et le contrôle radiologique était normal chez tous nos patients.

**Tableau 1:** Nature des corps étrangers

Nature du corps étranger	Nombre	Pourcentage (%)
Epingle à foulard	35	68,6
Punaise	4	7,8
Dent (incisive, molaire)	2	4
Prothèse dentaire	1	1,96
Cacahouète	1	1,96
Os olive	1	1,96
Haricot	1	1,96
Os de poulet (1,5/0,5cm)	1	1,96
Viande de poulet	1	1,96
Charbon	1	1,96
Seringue	1	1,96
Sonde gastrique	1	1,96
Canule de trachéotomie	1	1,96

## Discussion

L'inhalation accidentelle de corps étranger intrabronchique est particulièrement fréquente dans la première enfance, par rapport à l’âge adulte, avec une prédominance masculine dans les 2/3 des cas. Il s'agit d'une urgence diagnostique et thérapeutique. Elle constitue la localisation la plus redoutée en particulier chez l'enfant de moins de trois ans du fait de sa morbidité et sa mortalité qui varie de 0 à 0,7% selon les études [1–3]. Toutefois durant les deux dernières décennies, la fréquence des CE des voies aériennes chez l'enfant a considérablement baissé dans les pays développés du fait des compagnes de prévention et de sensibilisation [1].

L'incidence réelle de cette pathologie n′est pas connue car elle est souvent diagnostiquée tardivement ou le diagnostic est erroné. Ceci est plus fréquent chez les adultes que chez les enfants. Le taux est estimé à 0,66/100.000 habitants dans la population adulte aux États-Unis. Certaines statistiques avancent 100 cas par an pour les enfants contre deux cas par an pour les adultes chez qui l′incidence augmente après la quatrième décennie et atteint un pic à la septième décennie [4]. Dans notre série, les CE intrabronchiques prédominaient plutôt chez l'adulte jeune dont la moyenne d’âge était de 28 ans avec des extrêmes allant de 12 à 80 ans. Les facteurs qui influencent le risque d'inhalation de CE diffèrent selon l′âge. Chez l'enfant, la fréquence augmente dès l'acquisition de la préhension avec un pic autour de deux ans. Plus de 75,4% d'accidents d'inhalation surviennent chez l'enfant de moins de trois ans à l'occasion d'un jeu ou d'une prise alimentaire. Les corps végétaux devancent ceux d'origine inerte. Après l’âge de trois ans, lorsque la mastication devient plus efficace, leur fréquence diminue [1, 5].

Chez l'adulte, des études rétrospectives ont suggéré que les principales causes sont la diminution de la vigilance due à un état d'ivresse, une prise de sédatifs, une anesthésie générale, un traumatisme ou un handicap physique, un terrain de psychopathie, les émotions brutales au cours des repas et certaines professions (couturiers, cordonniers, menuisiers…).

Chez le sujet âgé, on note une prédisposition particulière aux fausses routes pendant le sommeil surtout à cause de la diminution du reflexe tussigène et de la déglutition en rapport soit avec la sénescence soit avec des maladies neurologiques ou neuromusculaires (Sclérose latérale amyotrophique, maladie d′Alzheimer, maladie de Parkinson’) soit avec certaines prescriptions médicamenteuses (anticholinergiques, antipsychotiques ou anxiolytiques). Aussi la tachypnée, associée à des conditions médicales communes à ce groupe d′âge, a montré une modification de la coordination entre la déglutition et la respiration. L'origine dentaire est aussi souvent incriminée (plombage, prothèses et chicots dentaires…) [2, 5–11].

Dans notre étude, les antécédents de maladies neuromusculaires s'observaient dans six cas (épilepsie, syndrome de Guillain barré et sclérose latérale amyotrophique), aussi deux cas de dépression, deux autres de scoliose, la trachéotomie après laryngectomie totale, le traumatisme facial et l'asthme dans un cas chacun.

La nature des CE inhalés dépend énormément des habitudes socioculturelles et éducatives des populations étudiées. Ainsi, elle serait dominée dans notre contexte et celui des pays islamiques par l'inhalation de l’épingle à foulard en rapport avec le port du voile, une coutume vestimentaire chez les jeunes adolescentes [3, 8, 9, 12]. L’étude de Zaghba et al a colligé une série de 26 cas d'inhalation d’épingle à foulard sur une période de près de 7 ans chez des jeunes marocaines de 12 à 30 ans [13]. Dans les séries Chinoises, ce sont les fragments osseux qui sont fréquemment retrouvés, chez les Vietnamiens le noyau de sapotier et au Moyen orient les pépins de melon [14].

L’élément clé du diagnostic est la survenue d'un syndrome de pénétration [15] qui se traduit par la survenue brutale chez un individu antérieurement sain d'une bradypnée inspiratoire et expiratoire, entrecoupée par des quintes de toux coqueluchoide avec parfois cyanose, tirage ou cornage. L'enclavement glottique est le risque majeur en cas de localisation trachéale pouvant même engager le pronostic vital à court terme [16]. Cependant, ce syndrome peut manquer ou être méconnu pendant des années responsable d'un retard diagnostique et d'une symptomatologie moins manifeste surtout chez l'adulte chez qui les CE ont tendance à s'enclaver dans les bronches distales. Ce constat rejoint celui de Nguyen et al [14] qui avaient colligé une série de 50 CE intrabronchiques enclavés surtout au niveau des lobaires inférieures dans 62% des cas. Dans la littérature, le syndrome de pénétration n′est retrouvé que dans moins de la moitié des cas (38%) [6, 17]. Chez nos patients, il a été relevé dans 90% des cas. Des hémoptysies peuvent émailler l’évolution et accroitre la suspicion de tumeur maligne surtout chez un sujet âgé ayant des facteurs de risque cancérigènes [5]. A noter que la présence d'un syndrome de pénétration impose une exploration endoscopique et ceci malgré un examen clinique et/ou radiologique normal ou non évocateur [1, 6]. Longtemps méconnu ou négligé, le CE peut entrainer des complications secondaires à son enclavement qui vont de l'emphysème obstructif à la pathologie infectieuse trainante ou récidivante toujours dans le même territoire (bronchopneumopathie infectieuse, abcédation, pleuropneumopathie, empyème, bronchectasie) en passant par les obstructions bronchiques totales (atélectasies), les réactions inflammatoires (granulomes). De rares cas de pneumomédiastin, de pneumothorax, d′emphysème sous-cutané ou de tableaux simulant une crise d'asthme ou une hernie diaphragmatique ont été rapportés dans la littérature [8, 10, 17–19].

La radiographie thoracique pourrait être d'un grand apport diagnostique en cas d'inhalation de CE avec une sensibilité et une spécificité respectivement de 68 à 73% et de 45 à 67% [20]. Ce constat n'est valable que pour les CE radio-opaques [7]; ce fut le cas chez 41 patients de notre étude. Pour les CE radio-transparents, non obstructifs ou de révélation encore précoce, les différents clichés seront normaux [13], et pour en améliorer la sensibilité des clichés en inspiration et expiration ou en décubitus latéral seront envisagés [1].

Selon plusieurs séries, le taux de négativité de l'imagerie chez les patients suspects d'inhalation de CE varie entre 5 et 30% chez l'enfant et entre 8 et 80% chez l'adulte, contre seulement 19,6% des cas dans notre étude [8, 9]. Ceci dit, certains signes radiologiques indirects comme l'atélectasie ou l'emphysème obstructif doivent alerter le clinicien [15, 7].

La localisation du CE varie selon les études. L'arbre bronchique droit est le siège le plus fréquemment retrouvé chez les adultes du fait de l'anatomie de la bronche principale droite qui prolonge la direction de la trachée [3, 13, 14, 20, 21, 22]. Comme cela a été illustré dans les séries de Caidi et al (53,3%) [5], Moura e Sá et al (61%) [4], Zaghba et al (69,2%) [13], et la nôtre (55,5%). Toutefois, la localisation gauche a été relevée dans plusieurs études, notamment celle de Diarra et al (76,92%) récemment publiée [22]. Un cas d'inhalation bilatérale de noix d'arec a été rapporté par Ur Rehman et al [23]. Alors que chez les enfants, il semble y avoir une répartition égale entre les arbres bronchiques droit et gauche [20].

Si le diagnostic clinique et radiologique est bien codifié, la stratégie thérapeutique pour l'extraction d'un CE trachéobronchique n′est pas encore consensuelle. Celle ci dépend étroitement de l'expérience des équipes [6]. Ceci dit la bronchoscope rigide sous anesthésie générale demeure la procédure de choix pour extraire les CE à tous les âges avec un taux de réussite de plus de 97% et une très faible morbidité et mortalité [24]. Actuellement, elle est parfaitement codifiée chez l'enfant comme c'est le cas dans la série pédiatrique de Mnejja et al [1]. Mais étant donné ses incidents potentiellement graves (0,96%), en particulier la désaturation, le laryngospasme, l'emphysème et l'oedème laryngé, certains auteurs proposent la bronchoscopie rigide en seconde intention après une bronchoscopie souple sous anesthésie locale à visée à la fois exploratrice et thérapeutique [1, 12, 25, 26]. Cette technique trouve particulièrement son indication chez les patients présentant des traumatismes cervico-faciaux, les patients ventilés mécaniquement, ou ayant un corps étranger de siège périphérique [4, 6]. Le taux de succès rapporté est supérieur à 90% [6, 26]. Il faut dire que ces deux techniques sont complémentaires et ne s′excluent pas mutuellement [4, 12].

Dans notre série, l'extraction était par bronchoscopie souple dans 40 cas et par bronchoscopie rigide dans seulement 3 cas. La chirurgie qu'elle soit radicale ou au mieux conservatrice peut être indiquée en cas d’échec de l'extraction endoscopique de CE vulnérants ou tranchants avec risque de migration ou de lésions vasculaires et aussi les CE méconnus avec destruction parenchymateuse irréversible [2, 4, 15, 22, 25]. Celle-ci serait préconisée dans 6 à 10,4% dans les pays en développement et dans 0 à 2,5% dans les pays développés où le délai de rétention est plus court et les moyens d'extraction plus adaptés [22]. C’était le cas chez quatre de nos patients. Certaines équipes utilisent même la thoracoscopie ou la cryothérapie comme moyen d'extraction [7, 14, 15].

## Conclusion

L'inhalation de CE intrabronchique est un accident rare chez l'adulte bien que sa prévalence augmente avec l’âge du fait de certains facteurs prédisposants. La présence d'un syndrome de pénétration, même en l'absence d'un examen radio clinique fortement évocateur, impose une exploration endoscopique à visée diagnostique et thérapeutique. La chirurgie d'extraction est une alternative finale en cas d’échec de celle ci ou de lésions parenchymateuses irréversibles. Ceci dit, dans notre contexte où l'inhalation d’épingle à foulard reste prédominante, la sensibilisation du public à travers les médias de masse a besoin d'attention pour diminuer le taux de croissance de cette situation.
